# Applying data mining techniques to determine important parameters in chronic kidney disease and the relations of these parameters to each other

**DOI:** 10.15171/jrip.2017.16

**Published:** 2016-11-20

**Authors:** Shahram Tahmasebian, Marjan Ghazisaeedi, Mostafa Langarizadeh, Mehrshad Mokhtaran, Mitra Mahdavi-Mazdeh, Parisa Javadian

**Affiliations:** ^1^Department of Health Information Management, School of Allied Medical Sciences, Tehran, University of Medical Sciences, Tehran, Iran; ^2^Department of Health Information Management, School of Health Management and Information Sciences, Iran University of Medical Sciences, Tehran, Iran; ^3^Department of Nephrology, Tehran University of Medical Sciences; Research Center of Iranian Tissue Bank, Tehran, Iran; ^4^Department of Internal Medicine, School of Medicine, Shahrekord University of Medical Sciences; Shahrekord, Iran

**Keywords:** Data mining in medicine, Parameter importance, Association rules, Chronic kidney disease

## Abstract

**Introduction:** Chronic kidney disease (CKD) includes a wide range of pathophysiological processes which will be observed along with abnormal function of kidneys and progressive decrease in glomerular filtration rate (GFR). According to the definition decreasing GFR must have been present for at least three months. CKD will eventually result in end-stage kidney disease. In this process different factors play role and finding the relations between effective parameters in this regard can help to prevent or slow progression of this disease. There are always a lot of data being collected from the patients’ medical records. This huge array of data can be considered a valuable source for analyzing, exploring and discovering information.

**Objectives:** Using the data mining techniques, the present study tries to specify the effective parameters and also aims to determine their relations with each other in Iranian patients with CKD.

**Material and Methods:** The study population includes 31996 patients with CKD. First, all of the data is registered in the database. Then data mining tools were used to find the hidden rules and relationships between parameters in collected data.

**Results:** After data cleaning based on CRISP-DM (Cross Industry Standard Process for Data Mining) methodology and running mining algorithms on the data in the database the relationships between the effective parameters was specified.

**Conclusion:** This study was done using the data mining method pertaining to the effective factors on patients with CKD.

**Implication for health policy/practice/research/medical education:**

The widespread use of medical information systems and the explosive growth of medical databases for become more efficient have had the needs to traditional data analysis using computer-assisted analysis. Data mining technique was used on the patients’ data to determine the weight and importance of the parameters.

## Introduction

Chronic kidney disease (CKD) is one of the most common diseases in the world. CKD includes a wide range of pathophysiological processes which will be observed along with abnormal function of kidneys and progressive decrease in glomerular filtration rate (GFR) (1). According to the definition decreasing GFR must have been present for at least three months. The different stages of this disease using KDOQI guide (kidney dialysis outcome quality initiative) is shown in Table 1 (2).

CKD is used to denote the processes in which there is irreversible decrease in nephrons number, is based on CKD stages 3 to 5. In order to classifying the stages of CKD, estimating GFR is essential. The normal range for GFR is from 120 mL/min/1.73 m^2^ which on average is lower in women than men. In CKD stages 1 and 2 the patients do not have any clinical symptoms attributable to CKD. If decreasing GFR progresses to stages 3, 4 the clinical and laboratory symptoms become significant and eventually will affect all of the vital organs. Yet the common symptoms include anemia, anorexia, malnutrition, disorders in the serum calcium levels, phosphorus and the regulating hormones of their hemostasis including parathyroid hormone (PTH), p 1.25 hydroxyvitamin D as well as disorders in sodium hemostasis, potassium, water and acid status (2).

It is estimated that at least 6% of adult Americans are in CKD stages 1 and 2, some unknown proportion of this population will progress to the higher stages. It is also estimated that 4.5% of Americans are in stages 3 and 4 (2).

CKD risk factors include the hypertension, diabetes, autoimmune diseases, African race, family history of kidney diseases, previous attacks of severe kidney injuries, protein in urine and the structural disease of urinary tracts (3).

In order to obtain new knowledge from among the vast array of data pertaining to patients with CKDs we can use data mining technology (4). Data mining appeared in 1990s and in this regard machine learning, database management and statistics have been used (5-7). Given the uses of electronic devices and also software solutions for patients’ data banks in medical sciences, huge amount of information are generated daily (7,8).

Considering the daily development of processing systems of computers and ever increasing power of computer processing system these two issues have allowed to discover knowledge from large data banks (7). The information age has activated many organizations to collect large amounts of information. Nevertheless, the data is useful only when the semantic information or new knowledge can be extracted from them (5,7,9). The increase in amounts of data has provided many business opportunities. It can be argued that, data mining and knowledge discovery from the databases is a new scientific field in engineering and computer sciences (7). Several definitions have been provided for data mining. The definition used in most of the references is “extracting information and knowledge and discovering hidden relations from the very large and complex databases” (5,6). Medical data is the most valuable and sensitive source for exploring and discovering, analyzing and obtaining knowledge (10). Therefore one of the most important majors in which data mining is used is the medicine. The increasing use of medical data systems and the ever increasing growth of medical databases need the analysis of traditional databases using the computers to improve the efficiency (11). The aim of this study is to find hidden relationships in a complete database of patients with CKD. There are mainly two basic purpose of data mining technology is predicted and described.

Using the whole data, the predictive data mining produces models for explaining the system which by applying them the variables performance can be predicted. Therefore the aim of predictive data mining is to make a model which by applying performance code, can do tasks such as prediction, ranking and estimation (10). The descriptive data mining based on the available data produces new information which explain behavioral models of the variables. The purpose of the descriptive data mining is to achieve to a thorough understanding of the system using the hidden models in it as well as the relations inside the data collections (6).

So far, there have been done many activities in discovering knowledge and the biological information of patients have been analyzed using the various data mining techniques and the relations and hidden models have been extracted from among the vast array of information (10,11).

## Objectives

By applying data mining the patients with CKD, this study tries to specify the factors most effective on this disease and also explain their relationship with each other.

## Materials and Methods

Considering the nature of this study which is the application of data mining in order to determine and analyze the data of patients with CKD, this study is data-oriented. The present study is mainly based upon discovering knowledge from the databases under study. Therefore the CRISP-DM (Cross Industry Standard Process for Data Mining**)** methodology has been used to do the research process (12). CRISP-DM provides an overview for mining projects. CRISP-DM steps include business understanding, data understanding, data preparation, modeling and evaluation. Activities during the study and based on the CRISP-DM methodology were described below.

### Data understanding

The statistical population of the study include 31996 data records registered in the database.

### Data preparation

First, all of the data has been tabulated with regard to the related field, then was analyzed by the conventional data mining software’s. These data include records containing personal information of each of the patients and also medical information of the patients with CKD. It has to be noted that these data include quantitative variables (including age and height) and also qualitative variables (Table 1). Likewise, the variables include numerical, ordinal and categorical types.

**Table 1:** CKD stages

**Stages**	**GFR (cc/min)**
0	>90
1	≥90
2	60-89
3	30-59
4	15-29
5	<15

### Modeling

After converting the data into the requested format of the software solutions and algorithms used in the research, the data was transferred to data mining software and then data mining techniques were applied on data. The algorithm applied to the data in this study is Association Rules. To apply the algorithms on the database, the data field Data Entry was done and then after the default phase some of the fields were removed and some of the fields were changed and during a number of interviews with the expert and checking the fields, we reached to the conclusion that the most effective data in this research is according to the Table 2.

**Table 2:** The fields list after pre-processing

** No **	**Parameter name**
1	Gender
2	Smoking
3	SBP (mm Hg)
4	Diastolic blood pressure (mm Hg)
5	Height (cm)
6	Weight (kg)
7	FBS (fast blood sugar) (mg/dL)
8	Cr (creatinine) (mg/dL)
9	Chol (Cholesterol) )(mg/dL)
10	HDL (high-density lipoprotein) (mg/dl)
11	LDL (low-density lipoprotein) (mg/dl)
12	TG (triglyceride) (mg/dl)
13	Hb (hemoglobin) (g/dL)
14	Hct (hematocrit)
15	MCV (mean corpuscular volume)
16	RBC (red blood cell count)
17	WBC (white blood cell count)
18	Protein (g/dL)
19	Age (years)
20	BSA (body surface area)(m2)
21	BMI (body mass index)
22	CKD-stage
23	MAP (mean arterial pressure) (mm Hg)

### Evacuation

In order to evaluate data mining results, rules and parameter importance were obtained from data compared with previous studies in CKD domain.

**Figure 1:**
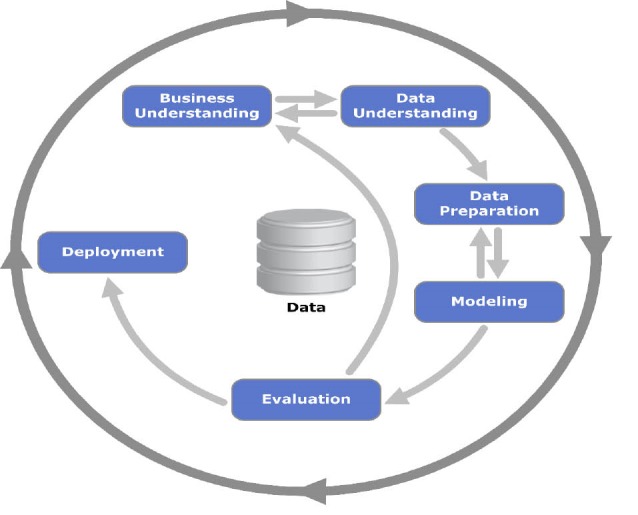
CRISP-DM methodology life cycle (12).

After applying CRISP-DM methodology on a dataset in health domain both valuable results in data and project documentation were obtained (Figure 1).

### Ethical issues

The research followed the tenets of the Declaration of Hel­sinki. To consider ethical issues in this research, all information fields related to patient identification were removed from the database before data mining process.

### Statistical analysis

Considering the registered information in the data bank we can obtain some relative comparisons and the frequency distribution which is tabulated as following (Tables 3 and 4):

**Table 3:** Relative frequency distribution of the sex of the patients under study

** Row **	**Sex **	**No. **	**Percent**
1	Male	31489	98
2	Female	507	2

**Table 4:** The relative frequency distribution based on CKD stages

** Row**	** CKD-stage **	**No. **	**Percent**
1	Stage-1	15288	47
2	Stage-2	13679	42
3	Stage-3	2007	6
4	Stage-4	10	0.5
5	Stage-5	4	0.2

## Results

Considering the application of different data mining algorithms to the available data, this process can be described in a number of rules as follows:

- **Rule1:** The relation of GFR with high-density lipoprotein cholesterol (HDL-C) increase simultaneously.
- **Rule2:** The relation of a number of variables with two phases of CKD phases:

In CKD stage 1 this relationships was observed; Hb (g/dL)>15.1, HDL-C (mg/dL) <42, FBS (mg/dL) <97.7, LDL-C (mg/dL) <132, BMI<22.8 (kg/m^2^)

In CKD Stage 2 this relationships was observed; Hb (g/dL) <14, HDL-C (mg/dL) >42, fasting blood sugar (FBS) (mg/dL) >97.7, low-density lipoprotein cholesterol (LDL-C) (mg/dL) >132.

**Rule3:** If (TG (mg/dL) >470), then (GFR (cc/min/1.73 m^2^) <70) with the possibility of 73%.

**Rule4:** Age is the most important factor in systolic blood pressure. But when systolic blood pressure >166 the distribution in different ages groups is almost the same.

**Rule5**: In CKD stage-1 the value of Hb (g/dL) is between 12 to14.

**Rule6**: With the mean arterial pressure (MAP) (mm Hg) parameter increase, the GFR (cc/min/1.73 m^2^) parameter usually decreases

**Rule7**: Persons with low body surface area (BSA) (m^2^) have probably low systolic blood pressure.

**Figure 2:**
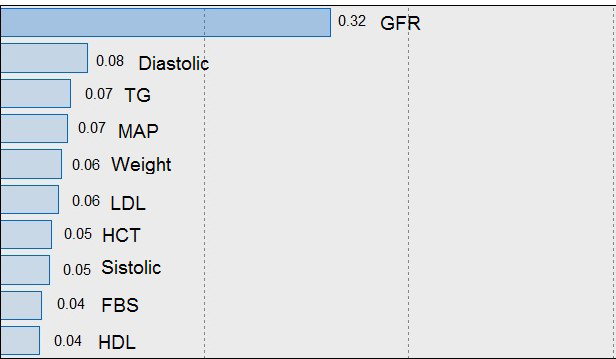
Parameter priority/importance funded in data mining process.

Also CKD parameter importance were calculated on the basis of analysis software. In the order of importance and significance of CKD variables are shown in Figure 2.

## Discussion

Each of the results of the data mining process were compared with clinical studies as follows:

The relations between GFR(cc/min/1.73 m^2^), HDL-C (mg/dL), LDL-C (mg/dL), and TG (mg/dL) were obtained in Rule1, Rule2 and Rule3 explained in some nephrology studies (13). The relation between HB(g/dL) and GFR(cc/min/1.73 m^2^) obtained in Rule2 confirmed in another study (14). The impact of age in blood pressure where obtained in Rule4 and The relation between MAP (mm Hg) and GFR (cc/min/1.73 m^2^) where obtained in Rule6 and the relation between BSA (m^2^) and systolic blood pressure were obtained in Rule7 approved in many studies (15). Most previous studies based on data mining techniques on patient’s data with kidney diseases used statistical or predictive methods. Results of previous studies have not included a CKD parameters order and CKD variable importance (10,16-18).

The rules obtained in this study and CKD parameter importance and weight can be considered as assumptions and evaluated in a clinical study.

## Conclusion

This study was done using the data mining method pertaining to the effective factors on patients with CKD and based on the literature so far there has not been done any study in this domain(4,19-22). Although according to the expert a limited number of CKD parameters have been used in this study. Thus, some new results have been extracted and unless another study is done on each of the discovered relations, the previous knowledge about CKD factors confirmed. If there were more effective fields available and they could be used in this study we could obtain more considerable results.

Considering the scope of the study, the following research contexts have been offered to the readers and researches:

- Comparing data mining results by the expert systems outputs to specify the factors having the highest risks on CKD.
- Offering rules by the data mining techniques including association rules model to specify the role of CKD factors.
- Using new data mining techniques including neural network and fuzzy model in order to predict the status of patients with CKD.

## Limitations of the study

A limitation of this study was the proportion of records and information fields in data set.

## Acknowledgments

We would like to thank Dr. Hormat Rahimzadeh, assistant professor of Department of Nephrology, SINA hospital in Tehran University Medical Sciences, for her excellent suggestions in data interpretation.

## Authors’ contribution

ST, MG and ML; participated in all experiments, coordinated the data-analysis and contributed to the writing of the manuscript. MM; coordinated the acquisition of data. PJ and MM; designed the research plan and organized the study. PJ; performed analysis and interpretation of data.ST; prepared the final manuscript.

## Conflicts of interest

The authors declare no conflict of interest.

## Funding/Support

This research has been supported by Tehran University of Medical Sciences (Grant# 25350).

## Ethical considerations

Ethical issues (including plagiarism, data fabrication, double publication) have been completely observed by the authors.
